# Compromised future thinking: another cognitive cost of temporal lobe epilepsy

**DOI:** 10.1093/braincomms/fcac062

**Published:** 2022-03-19

**Authors:** Genevieve Rayner, Mariana Antoniou, Graeme Jackson, Chris Tailby

**Affiliations:** 1 Florey Institute of Neuroscience and Mental Health, Heidelberg, VIC, Australia; 2 Melbourne School of Psychological Sciences, The University of Melbourne, Parkville, VIC, Australia; 3 Department of Clinical Neuropsychology, Austin Health, Heidelberg, VIC, Australia

**Keywords:** temporal lobe epilepsy, cognition, future thinking, prospection, epilepsy

## Abstract

The ability to mentally travel forward through time allows humans to envisage a diverse array of possible events taking place in the future, helping us to choose which pathway to take in life. In epilepsy, we assume that patients use this cognitive ability when deciding between various treatment options, but this assumption has not been robustly tested. The temporal lobes are key contributors to this ‘future thinking’ and its building blocks include cognitive functions commonly impaired in temporal lobe epilepsy such as memory and language, giving rise to a hypothesis that ‘future thinking’ is impaired in this patient cohort. Participants were 68 adults: 37 with neurosurgically-naïve, unilateral temporal lobe epilepsy (51% right lateralized) and 31 healthy controls of similar age, sex and intellectual ability to the participants with epilepsy. Future thinking was measured using an imagined experiences task validated in other neurological populations. Tools well-established in temporal lobe epilepsy were used to measure potential cognitive correlates of future thinking. Analysis of variance revealed significantly impoverished future thinking in both left and right temporal lobe epilepsy relative to controls (*P* = 0.001, *η*_p_^2^=0.206), with no difference between temporal lobe epilepsy groups (*P* > 0.05). Future thinking deficits in left temporal lobe epilepsy were paralleled by deficits in scene construction, whereas impoverished future thinking in right temporal lobe epilepsy occurred in the setting of intact scene construction. Deficits in future thinking were associated with reductions in lexical access and episodic autobiographic memory in both epilepsy groups. In sum, future thinking is compromised in both left and right temporal lobe epilepsy. The deficit in left temporal lobe epilepsy is largely explainable by dysfunction in verbal cognitive processes including scene construction. While the basis of the deficits observed with right temporal foci shares features with that of left temporal lobe epilepsy, their intact scene construction raises questions about the role of the left and right temporal lobes in future thinking and scene construction and the relationship between these two constructs, including whether right temporal lobe might play a specific role in future thinking in terms of creative processing. Clinicians should take impaired future thinking into account when counselling temporal lobe epilepsy patients about various treatment options, as they may struggle to vividly imagine what different outcomes might mean for their future selves.

## Teaser

Rayner *et al*. report that patients with left and right temporal lobe epilepsy (TLE) demonstrate clinically significant impairments in future thinking ability relative to healthy controls. This result has important implications for how clinicians inform and counsel patients with TLE during treatment decision-making.

## Graphical Abstract

**Graphical Abstract:**
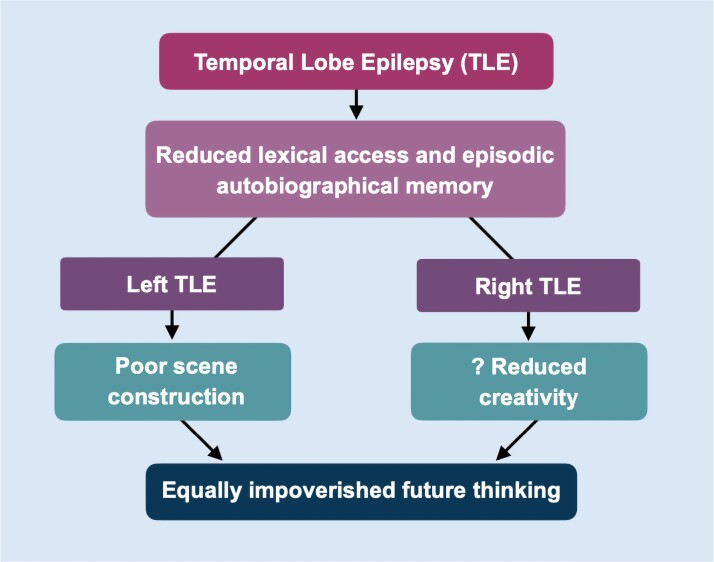

## Introduction

The presurgical counselling of focal epilepsy patients typically assumes that they can mentally project themselves into an imagined future, envisaging the potential impacts of different treatment outcomes on their lifestyle.^1,2^ This future thinking requires richly detailed mental simulation of multiple hypothetical future scenarios, for instance:

“What will life look like if I have surgery and become seizure-free?

…How will things change if I get my licence back?

…What happens if my memory or word finding deteriorates?

…Will I still be able to work?”

A growing body of literature has shown that future thinking is a multidetermined or ‘higher-order’ cognitive act. It draws upon a number of cognitive systems to reshape previous experiences and knowledge into novel simulations of the future; these include systems such as autobiographic memory, semantic knowledge, scene construction, mental visualization and spatial processing, emotion, theory of mind, planning and cognitive control.^3^ Together, these cognitive systems form the necessary building blocks from which future thinking emerges. From these cognitive foundations, future thinking allows humans to engage in the extraordinary ability to mentally travel forward through time so that they might plan which pathways to take in life so as to achieve their personal goals. Its ‘higher-order’ nature is reflected in the observation that many neurocognitive disorders can have the common feature of impoverished future thinking, despite their heterogeneous aetiologies and cognitive profiles; these include Alzheimer’s disease, dense amnesias, semantic dementia, behavioural variant frontotemporal dementia, Parkinson’s disease, and major depression.^4–9^ The unique qualia associated with reflecting on one’s personal future was emphasized by Tulving, who introduced the concept of *proscopic chromesthesia* (future-orientated mental time travel) to capture this. This shift in conscious experience distinguishes future thinking from its lower-order cognitive aspects, and speaks to its status as a distinct cognitive function.^10^

Functional neuroimaging studies of future thinking ascribe particular importance to the temporal lobes.^11,12^ Both mesial and lateral temporal structures seem to be key hubs in a bilateral neurocognitive network that also involves mesial prefrontal cortex, precuneus and retrosplenial cortices.^13,14^ This distributed system resembles networks involved in autobiographical recollection,^14–16^ likely reflecting how our past experiences shape how we imagine the future.^17^ Despite it being well-established that people with temporal lobe epilepsy (TLE) are vulnerable to cognitive dysfunction across many of the domains implicated in prospection^18,19^ as well as to neurobiological dysfunction in the temporal lobe, future thinking remains little studied in TLE.

In the present behavioural study, we aimed to investigate the effect of unilateral TLE on prospection ability. An initial investigation by Lechowicz *et al*.^20^ reported that a mixed group of pre- and post-surgical unilateral TLE patients produced fewer details related to place, time and emotion than healthy controls when simulating future events. Left and right TLE were equally poor at this aspect of future simulation, although people with left TLE did generate significantly less ‘perceptual’ details in their description of future events. The study was, however, limited by small sample sizes (*n* = 10 per group) that likely underpowered statistical analyses, as well as the undifferentiated inclusion of both pre- and post-surgical cases obscuring whether deficits are a consequence of resection or are present preoperatively. This distinction is of central importance to clinical practice, as evidence of preoperative difficulties in future thinking would have significant implications for the process of presurgical counselling.

Here we investigate whether people with neurosurgically naïve, drug-resistant, unilateral TLE show diminished future thinking relative to controls in the largest and most homogeneous sample to date. Further, we explore potential laterality effects by contrasting the future thinking performances of left and right TLE patients. Improving on previous methodologies, we use a measure of prospection that separates future-orientated thinking from broader episodic scene construction, visualization and description.^21,22^ Lastly, we conduct exploratory analyses examining cognitive and clinical factors that contribute to future thinking ability, as a means of identifying potential drivers of dysfunction that could inform approaches to future-orientated patient counselling.

## Materials and methods

### Participants

Inclusion criteria for all participants were: (i) aged 18–70, (ii) intelligence quotient (IQ) estimated as ≥70, (iii) no history of neurosurgical intervention, (iv) functional English. The latter was established clinically; specifically, participants who were non-native English speakers and required either an interpreter or additional language aids during clinical conversations or psychometric testing were not recruited to the study. IQ was estimated using the Test of Premorbid Functioning,^23^although in cases where a developmental dyslexia was diagnosed (*n* = 7; four with left TLE and three right TLE), IQ was estimated using perceptual indices of intellectual capability from the Wechsler Abbreviated Scale of Intelligence-II.^24^ Exclusion criteria for patients comprised a history of neurological disease other than epilepsy, and current comorbid psychiatric diagnoses other than mild–moderate anxiety or unipolar mood disorder. Exclusion criteria for the controls comprised any history of neurological disease or injury, and current comorbid psychiatric diagnoses other than mild anxiety or unipolar mood disorder. Psychiatric screening was established via the Structured Clinical Interview for DSM-IV Axis I Disorders.^25^

Individuals who met the pre-established inclusion/exclusion criteria were prospectively recruited to the cross-sectional study, with all 78 individuals who consented participating. Standard cleaning of the dataset resulted in the removal of 10 of these initial participants: two healthy controls who performed as outliers (*z* < −2) on the future thinking task; one additional healthy control with incomplete cognitive data; five people with left TLE and incomplete cognitive data and two right TLE with incomplete cognitive data. As such the final dataset comprised 68 adults, with *post hoc* analysis confirming that this sample size has sufficient power to detect medium–large effect sizes at the 0.95 level in a multiple regression. Participants who were excluded for incomplete data did not significantly differ from the remaining participants on any clinical or demographic variables (*P* < 0.05).

The patient sample (*n* = 37) was recruited while undergoing inpatient video-EEG characterization of focal seizures in the Comprehensive Epilepsy Program of Austin Health, Melbourne. Epileptogenic foci were localized and lateralized to the temporal lobe by well-established methods published by this group,^26,27^ including clinical history, ictal semiology, video-EEG monitoring, 3-T structural MRI, interictal [18F]fluorodeoxyglucose PET, ictal and interictal blood flow single-photon emission computerized tomography, and clinical neuropsychological evaluation. Eighteen TLE patients had foci lateralized to the left hemisphere and 19 had right TLE; demographic and clinical features of the patient samples are summarized in Table 1. There were no significant differences between right and left TLE patients in age at seizure onset, duration of epilepsy, seizure frequency or number of medications (*P* > 0.05). There were also no significant differences between left and right TLE groups with respect to lesion status, intratemporal localization or polytherapy status (*P* > 0.05).

**Table 1:** Demographic and clinical profiles of TLE groups and healthy controls

	Left TLE (*n* = 18)	Right TLE (*n* = 19)	Healthy controls (*n* = 31)
Age (years), *M* ± SD	36.56 ± 12.67	39.79 ± 12.99	43.97 ± 12.37
Range	20–58	18–65	19–66
Sex female (%)	8 (44%)	11 (58%)	19 (63%)
Education (years), *M* ± SD	13.11 ± 2.72	14.24 ± 3.78	14.58 ± 3.77
Range	8–18	9–23	9–25
Estimated IQ, *M* ± SD	97.11 ± 9.69	102.89 ± 10.03	102.94 ± 10.41
Range	79–117	79–114	81–126
Current affective disorders			
Any depressive disorder	2 (11%)	4 (21%)	6 (19%)
Any anxiety disorder	7 (39%)	5 (26%)	11 (35%)
Age seizure onset (years), *M* ± SD	19.48 ± 12.86^a^	23.05 ± 16.11	
Range	0.3–55	0–59	
Duration epilepsy (years), *M* ± SD	16.79 ± 13.76^a^	16.37 ± 16.02	
Range	2–52	2–62	
Monthly seizure frequency			
*M* ± SD	22.44 ± 56.39	18.12 ± 28.40	
Median	4.50	6.00	
Range	0–240	0–112	
Lesion status			
Lesion resolvable on MRI	12 (66%)	13 (72%)	
No lesion on MRI	6 (33%)	5 (28%)^a^	
Intra-lobe localization			
Mesial^b^	7 (39%)	6 (32%)	
Lateral	6 (33%)	6 (32%)	
Uncertain	5 (28%)	7 (37%)	
AED polytherapy (%)	15 (83%)	11 (58%)	
Number AEDs, *M* ± SD	2.33 ± 0.91	2.21 ± 1.27	
Range	1–4	1–5	

TLE, temporal lobe epilepsy; IQ, intelligence quotient; AEDs, antiepileptic drug.

^a^ There was one case of missing data.

^b^ Includes cases with some lateral involvement in addition to a mesial focus (three LTLE, one RTLE).

A group of 31 healthy individuals recruited from the patients’ families provided a sociodemographically matched control sample. Patients and controls were tested separately and asked not to discuss their participation in order to avoid cross-contamination. There were no significant differences between right TLE patients, left TLE patients and healthy controls for estimated IQ [*F*(2,65) = 2.179, *P* = 0.121], years of education [*F*(2,65) = 1.01, *P* = 0.37], sex (*χ*^2^ = 1.354; *P* = 0.51) or age [*F*(2,65) = 2.055, *P* = 0.136]; see Table 1.

### Cognitive assessment

#### Future thinking

The scene construction task^4,28^ was selected to measure future thinking capability, as it is designed to gauge participants’ ability to vividly imagine events in the mind’s eye and verbally describe them (*scene construction*) separately from their ability to project themselves into imagined future events (*episodic future thinking*). Although both scene construction and future thinking involve imagining an experience from an egocentric viewpoint, scene construction tasks are not explicitly temporal in nature nor do they draw upon the imaginer’s self-schema and personal goals in the same way thinking about a plausible personal future event does.

We used an abbreviated version of the task comprising six imaginary scenarios that the participants are asked to cast themselves in and describe aloud; the first three elicit a rich description of a fictitious scene (scene construction; e.g. ‘*Imagine you’re sitting having a drink in a pub. I want you to describe the experience and the surroundings in as much detail as possible using all your senses including what you can see, hear and feel, as well as what you are those around you are doing*') with the other three designed to project the participant into an imaginary future scenario (episodic future thinking; e.g. ‘*Imagine the next time you’ll meet a particular friend you haven’t seen for a while. But just give me one event, what we’re looking for is a single scene, a snapshot moment. I want you to describe that scene and the surroundings in as much detail as possible using all your senses including what you can see, hear and feel, as well as what you and your friend are doing*'). Participants are explicitly told not to describe a remembered event, but to create something new. Prompts were given as per the original protocol.^4^ There was no time limit imposed on participants.

Responses were recorded and later transcribed for scoring as per the scoring system in the original publication^28^ (a full description of the scoring is contained in the Supplementary material of the original paper by Hassabis *et al.*). In brief, a composite score was used to index the overall richness of each imagined scenario, ranging from 0 to 60 (0 = not experienced at all, 60 = extremely richly experienced); this included scores for the level of detail in the content (*T*-scores), spatial coherence (SC score), the rater’s opinion on the scenario’s overall evocative and experiential qualities (*Q*-score) and the participant’s subjective rating of the scenario’s vividness (*S*-score) as well as how much of a sense of presence or being there they felt while imagining (*P*-score). Scores were summed across the three scene construction scenarios and across the three future thinking scenarios. Interrater reliability on the scene construction task has been formally estimated at *α* = 0.91^29^; nonetheless for the current study, the assessors (M.A. and G.R.) undertook regular interrater scoring monitoring where the alternate scorer was blind to diagnosis where possible (for instance, providing that the simulations did not contain details identifying of participant status), with a consensus style approach taken.

#### Factors contributing to future thinking

Tests measuring cognitive factors potentially contributing to future thinking are summarized in Table 2. Tests were typically administered in the following order: scene construction task; learning trials of memory tests; executive and language tests; delay trials of memory tests; autobiographical memory interview.

**Table 2:** Tests measuring cognitive factors contributing to future thinking ability in TLE

Test	Critical metric	Task description
Autobiographical memory test^30^	Personal episodic and semantic memory	The Personal Semantic Schedule requires participants to recall personally relevant facts (e.g. former addresses). The Autobiographical Incident Schedule asks participants to recall three episodes from each time period (e.g. a wedding).
Rey Auditory-Verbal Learning Test^31^	Auditory-verbal new learning and recall	Participants are required to acquire and recall a list of 15 nouns (List A) over five learning trials, with free recall of these words demanded following an interference trial of an alternate list of 15 nouns (List B), and again following a 30-min delay.
Controlled Oral Word Association Test^31^	Orthographic and semantic lexical retrieval	Task requires participants to orally produce as many words as possible beginning with a specified letter (i.e. F, A and S), within a 1-min interval. The ‘Animals’ subtest requires participants to orally produce as many animal names as possible within a 1-min interval
Boston Naming Test-2^32^	Visual confrontation naming	Task comprises 60 black-and-white line drawings of common objects that participants are required to name aloud. The objects gradually increase in difficulty and range from simple, high-frequency vocabulary words (e.g. *comb*) to rare words (e.g. *abacus*).
Digit span backwards^23^	Cognitive control	Task requires participants to hold increasing lengths of digits in their working memory and repeat them back to the examiner in the reverse order.

### Statistical analyses

Analyses were performed using IBM SPSS Statistics, with significance set at *P* < 0.05 (two-tailed) and corrected for multiple comparisons where appropriate. Where data did not meet assumptions for parametric analyses, non-parametric alternatives were employed e.g. Mann–Whitney *U*, Brown–Forsyth. For categorical variables employing *χ*^2^ analysis, exact significance values are reported (two-sided), with continuity corrections in the case of 2 × 2 analyses. Raw test scores were used in the analyses given that systematic, robust normative data stratified by age were not available for all tools; moreover, assessing patient scores relative to a normative sample was the purpose of the healthy control group.

One-way between-subjects ANOVA tests were employed to detect any differences between left TLE, right TLE and healthy controls on the measure of future thinking, with planned comparisons used to test the hypothesis that patients with left and right TLE would have diminished future thinking ability relative to healthy controls. This process was repeated to look at patterns on the future thinking subscales of the scene construction task. Analyses were then re-run using the clinician’s judgement of participants’ future thinking aptitude (i.e. ‘*Q*-score’ from the scene construction task) as the dependant variable, to explore the notion of whether the expert clinical evaluation of future thinking ability is a simple yet valid way of assessing this cognitive function.

We then sought to examine whether any between-group differences in future thinking ability were paralleled by differences in scene construction capacity. A mixed two-way ANOVA was conducted with effects of task (two levels: future thinking, scene construction) and group (three levels: left TLE, right TLE, controls).

Finally, correlation analyses were used to evaluate whether variation in future thinking among patients and controls is related to variation in theoretically related cognitive abilities (such as verbal learning and recall, autobiographic memory, verbal capability and cognitive control). Correlations between future thinking and these variables were calculated separately for each group (left TLE, right TLE, control), and correlations were compared based on their 95% confidence intervals. If no between-group differences in *r* were present, the correlation between a given cognitive variable and future thinking was calculated across the whole sample; otherwise group-specific *r* values were retained.

Randomization was not required given the design of the study did not include any intervention; blinding was not feasible given that participants were recruited and tested on a video-EEG ward, meaning that patients were easily distinguishable from controls.

### Standard protocol approvals, registrations and patient consents

The study had approval from the Human Research Ethics Committees where the work was conducted, i.e. Austin Health and The University of Melbourne, and all participants provided written, informed consent in accordance with the Declaration of Helsinki.

### Data availability statement

In the interests of data transparency, the authors will provide interested colleagues with the raw, de-identified data on request.

## Results

### Future thinking is impaired in TLE

**Figure 1:**
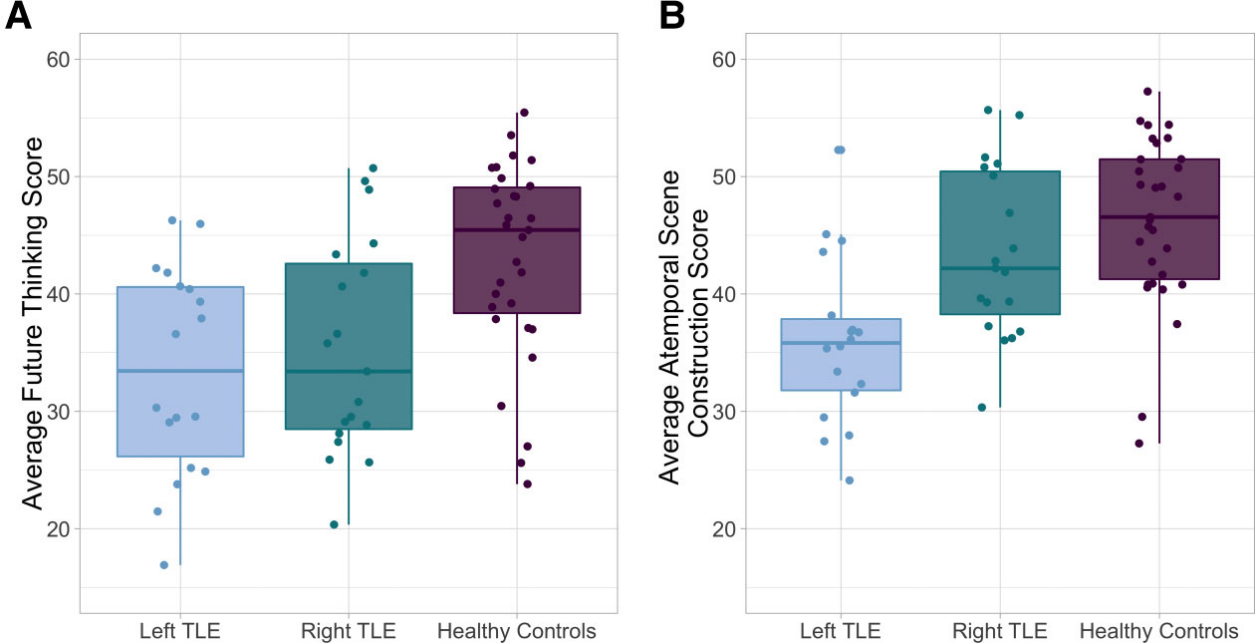
**Future thinking is impaired in both left and right TLE, but scene construction varies by group.** (**A**) One-way ANOVA on average scores across the three trials of the future thinking element of the task shows that future thinking is impaired in both left and right TLE relative to healthy controls, *F*(2,65) = 8.412, *P* = 0.001, *N* = 68. (**B**) ANOVA on average scores across the three trials of the scene construction element of the task shows a main effect of group on scene construction performances, *F*(2,65) = 12.1, *P* < 0.001, with planned comparisons revealing that patients with left TLE performed worse than patients with right TLE (*t*_(35)_ = −3.232, *P* = 0.002) and controls (*t*_(47)_ = −4.888, *P* < 0.001).

A one-way ANOVA addressing the principal question of whether future thinking is impaired in TLE showed that the scores of the three groups differed significantly on the future thinking subtest of the scene construction task, *F*(2,65) = 8.412, *P* = 0.001, *η*_p_^2^ = 0.206, i.e. large effect size (see Table 3). Planned comparisons revealed that patients with left TLE (*t*_(47)_ = 3.701, *P* < 0.0001) and right TLE (*t*_(48)_  = −3.023, *P* = 0.004) performed significantly worse than controls; there was no significant difference between left and right TLE (*t*_(35)_  = −0.656, *P* = 0.514; Fig. 1A). This same pattern of results held when the clinician’s judgement of future thinking (total *Q*-score) was used as the dependent variable [*F*(2,65) = 9.51, *P* < 0.001; correlation between total future thinking composite and total *Q*-scores is *r* = 0.91].

**Table 3:** Cognitive profile of TLE subgroups versus healthy controls

	Left TLE (*n* = 18)	Right TLE (*n* = 19)	Healthy controls (*n* = 31)	Effect size *η*_p_^2^
*Future thinking—scene construction task*				
Future thinking, *M* ± SD	33.4 ± 8.90	35.3 ± 9.14	43.0 ± 8.32***	0.206
Scene construction, *M* ± SD	36.0 ± 7.05	43.5 ± 7.22	46.3 ± 7.08***	0.271
*Relevant Cognitive Domains*				
BNT-2, *M* ± SD	49.83 ± 6.46	50.53 ± 8.98	55.13 ± 4.23**	0.134
COWAT (animals), *M* ± SD	20.94 ± 5.21	20.95 ± 6.54	24.29 ± 6.65	0.069
COWAT (FAS), *M* ± SD	30.06 ± 11.07	35.37 ± 12.66	43.52 ± 13.91**	0.170
Digits backward, *M* ± SD	4.50 ± 1.465	4.68 ± 1.003	5.39 ± 1.498	0.082
AMI episodic recall, *M* ± SD	18.67 ± 5.53	19.16 ± 4.24	22.00 ± 3.44*	0.119
AMI semantic recall, *M* ± SD	54.75 ± 5.89	56.63 ± 3.79	57.74 ± 4.42	0.066
RAVLT ∑A1–5, *M* ± SD	49.72 ± 8.04	53.11 ± 8.30	58.84 ± 8.47	0.186
RAVLT A7, *M* ± SD	10.35 ± 2.55	10.89 ± 3.56	11.84 ± 3.71	0.035

N.B. all scores are raw scores; interpretation of effect sizes is as follows—small = 0.01, medium = 0.06, large = 0.14. BNT, Boston Naming Test; COWAT, Controlled Oral Word Association Test; AMI, autobiographical memory interview.

* *P* < 0.05; significance tests refer to overall *F*-tests from one-way ANOVA.

** *P* < 0.01; significance tests refer to overall *F*-tests from one-way ANOVA.

*** *P* < 0.001; significance tests refer to overall *F*-tests from one-way ANOVA.

As expected, given the matching of groups for intellect, the pattern of results remained unchanged when IQ was included as a covariate (effect of group: *F* = 7.3, *P* = 0.001, left TLE versus control: *P* = 0.007, right TLE versus control: *P* = 0.01, left TLE versus right TLE, *P* = 0.96). The results were similarly unchanged when analyses were restricted to the subset of patients with no known dyslexia (effect of group: *F* = 6.6, *P* = 0.003, left TLE versus control: *P* = 0.007, right TLE versus control: *P* = 0.04, left TLE versus right TLE, *P* = 0.79).

Secondary analysis of the subscale scores for the future thinking aspect of the scene construction task showed that both left and right TLE are worse than controls on the content scale (*P* < 0.01 in each case) and the quality scale (*P* < 0.001 in each case); and that left TLE is worse than both right TLE and controls on the SC scale (*P* = 0.03 in each case) with right TLE and controls comparable to one another. There were no group differences on metrics of subjective salience or presence (i.e. sense of reexperiencing; see Supplementary material for a summary of this pattern of results). Together, these analyses indicate that people with TLE do worse on the future thinking task because of poor scores on the content and quality scales, with left TLE also producing poor SC scale scores.

### Future thinking linked to poor scene construction in left TLE

Having demonstrated that comparable deficits in future thinking are present in the left and right TLE, we then examined whether this might simply reflect a reduced capacity for atemporal narrative scene construction in TLE. To this end, we conducted a mixed two-way ANOVA, with effects of task (two levels: future thinking, scene construction) and group (three levels: left TLE, right TLE and control). The interaction was significant [*F*(2,65)  = 6.6, *P* = 0.002, *η*_p_^2^ = 0.022, i.e. small effect size], indicating that the effect of task differed across the groups (Fig. 1B). We have already shown that future thinking was compromised to a comparable degree in both TLE groups relative to controls. Follow up one-way ANOVA on the scene construction task showed a main effect of group, *F*(2,65) = 12.1, *P* < 0.001, *η*_p_^2^ = 0.271, i.e. large effect size (see Table 3). Planned comparisons revealed that patients with left TLE performed worse than patients with right TLE [*t*(35)  = −3.232, *P* = 0.002] and controls [*t*(47)  = −4.888, *P* < 0.001], with scores of right TLE and controls not differing [*t*(48) = −1.323, *P* = 0.19].

Thus, while left TLE performed worse than controls on both future thinking and scene construction, only future thinking was affected in right TLE; their scene construction ability was comparable to controls and significantly better than left TLE (Fig. 1). This suggests that while the reductions in future thinking observed in left TLE likely relate to reductions in scene construction, this same explanation cannot account for the reduction in future thinking in right TLE.

### Poor future thinking in TLE is associated with reduced lexical access and autobiographic memory

In an effort to understand the basis of the compromised future thinking in TLE, we conducted exploratory analyses to evaluate whether variation in future thinking among patients and controls is related to variation in cognitive abilities such as verbal learning and recall, autobiographic memory, verbal capability and cognitive control (see Tables 2 and 3). We examined this in two ways: (i) using univariate between-group ANOVA to see if performances on these cognitive tasks varied between the groups and (ii) by looking at the correlation of those same variables with future thinking scores.

Considering first the univariate between-group effects, people with left and right TLE both performed significantly worse than the healthy controls on measures of confrontation naming [Boston Naming Test (BNT)-2], orthographic lexical retrieval [Controlled Oral Word Association Test (COWAT)-FAS] and episodic autobiographical memory interview (AMI_ep_), with no difference between the two TLE groups on any measure (*P* > 0.05). Examination of effect sizes revealed that atemporal scene construction has the largest effect size (*η*_p_^2^ = 0.271), followed closely by future thinking (*η*_p_^2^ = 0.206; i.e. very large effect sizes). The magnitude of effect sizes in the more commonly used psychometric tests ranged from large [*η*_p_^2^=0.186; Rey Auditory-Verbal Learning Test (RAVLT) ∑A1–A5] to small–medium (*η*_p_^2^=0.035; RAVLT trial A7 recall). These additional univariate analyses suggest that reduced future thinking in both right and left TLE might stem, at least in part, from difficulties with lexical access (BNT-2, COWAT-FAS) and AMI_ep_.

In line with the univariate findings, the correlation analyses reported in Table 4 also show that across the whole sample, future thinking was associated with measures of lexical access (BNT-2, COWAT-Animals, COWAT-FAS), executive function (COWAT-FAS) and AMI_ep_. Semantic AMI_sem_ were also correlated with total future thinking scores in controls, but not in the right or left TLE groups. Anterograde memory (RAVLT indices) and working memory (digits backwards) were not significantly correlated with total future thinking scores.

**Table 4:** Correlations between future thinking and theoretically related cognitive variables

	FT: whole sample	FT: left TLE	FT: right TLE	FT: control
*BNT*	**0.42**			
*Animals*	**0.43**			
*FAS*	**0.36**			
*LDSB*	a	*0.43*	−*0.14*	−*0.16*
*RAVLT_sum_*	*0.21*			
*RAVLT_delay_*	*0.08*			
*AMI_auto_*	**0.35**			
*AMI_sem_*	b	−*0.03*	−*0.04*	**0.46**

Correlations were initially calculated separately for each group. If no significant (*P* < 0.05) differences in *r* were observed between any of the groups then *r* for the whole sample (collapsed across group) is reported; otherwise, group level *r* values are reported. Significant *r* values (*P* < 0.05) are shown in bold; non-significant *r* values (*P* > 0.05) are shown in italic. FT, future thinking; BNT, Boston Naming Test; Animals: animal fluency; FAS, letter fluency; LDSB: longest digit span backwards; AMI_auto_, episodic score from the autobiographical memory interview; AMI_sem_, semantic score from the autobiographical memory interview.

^a^ *r*_left TLE_ > *r*_right TLE_ and *r*_left TLE_ > *r*_control_.

^b^ *r*_left TLE_ < *r*_control_ and *r*_right TLE_ < *r*_control_.

## Discussion

There are two major outcomes from the current study. Firstly, both left and right neurosurgically naïve TLE patients show significant dysfunction in their future thinking capability relative to healthy controls, with large effect sizes suggesting that as a group they perform in the lowest 15th percentile of the population. This cognitive deficit in epilepsy has important clinical implications for how we counsel patients, raising questions about how we might improve the process of informing and supporting people with TLE during treatment decision-making.

**Figure 2:**
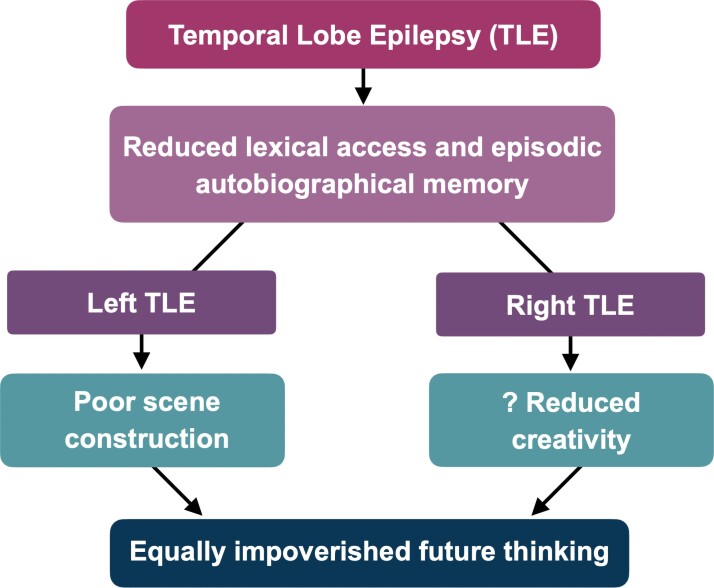
**Model of impaired future thinking in left versus right TLE.** Future thinking is compromised in both left and right TLE [*F*(2,65) = 8.412, *P* = 0.001]. In both groups, deficits in future thinking were associated with reductions in lexical access (*r* = 0.36–0.45) and episodic autobiographic memory (*r* = 0.35). Future thinking deficits in left TLE were paralleled by deficits in scene construction [*F*(2,65) = 12.1, *P* < 0.001], whereas, impoverished future thinking in right TLE raises the question as to whether right temporal lobe might play a specific role in future thinking in terms of creative processing.

Second, the comparable deficits in future thinking observed in left and right TLE seem to arise through common reductions in lexical access and autobiographic memory in both groups, along with a scene construction deficit in left TLE (see Fig. 2). This latter observation begs the question of why future thinking in the left TLE group is not even worse than right TLE; or expressed conversely, why future thinking is so notably compromised in right TLE given intact scene construction? The role of the left and right temporal lobes in future thinking and scene construction is discussed, together with the potential relationship between these two constructs.

### Basis of impaired future thinking in left TLE

Future thinking is conceived of as a higher-order cognitive act, drawing upon multiple other cognitive processes. This conceptualization is reflected in our data, which showed that across both epilepsy and control groups lexical retrieval processes and episodic aspects of autobiographic memory (but not working memory nor auditory-verbal memory) are associated with variance in future thinking.

Beyond these common cognitive factors, the impoverished future thinking of people with left TLE specifically was also associated with reduced scene construction capability (see Fig. 2). These findings are broadly consistent with Lechowicz *et al.*’s^20^ observation that people with left TLE provide significantly less perceptual details in their descriptions of future events relative to right TLE and healthy controls, i.e. their evocation and description of the future were particularly lacking in the visual, olfactory, gustatory, auditory and tactile details that build the sensorium of a scene. Although intact scene construction does not guarantee intact future thinking (*see* our right TLE patients), this finding clearly underscores that scene construction is a crucial aspect of being able to simulate and verbally describe richly detailed scenarios centred around a future version of oneself.

Notably, the current findings are in contrast to those showing that people with semantic dementia demonstrate intact scene construction^33^ and intact autobiographical recollection^8^ together with striking impairments in simulating scenarios involving their possible future selves.^8^ Semantic dementia is a clinical condition that reflects progressive degeneration of the anterior temporal lobes, typically lateralized to the left. The double hit (poor scene construction + future thinking) seen in our cohort of patients with left temporal lobe disease may speak to the greater involvement of the mesial temporal structures in left TLE,^34^ compared with the typically more neocortical predilection of semantic dementia^35^ pathology. The significance of the relationship between scene construction and future thinking in TLE is examined further below.

### The contribution of the right temporal lobe to future thinking

Reduced future thinking was also present in the right TLE group. It has been argued previously that scene construction, a crucial element of future thinking, may be dependent on the right temporal region.^33^ One might have therefore expected future thinking *and* scene construction to be impaired in the right TLE group. Our data do not support this prediction: narrative scene construction and SC scores were normal in the right TLE group, demonstrating that scene construction can remain intact in the presence of right temporal lobe disease. This is consistent with cases studies of developmental^36^ or adult-acquired^37^ bilateral hippocampal damage that similarly show normal scene construction in the presence of right temporal disease. Moreover, contrary to the hypothesis of a special role for the right temporal lobe in scene construction, our results show that scene construction is more likely to be impaired in the presence of unilateral *left* TLE. Our findings in right TLE also highlight that scene construction and future thinking are dissociable constructs, as has also been reported in the setting of prefrontal lesions,^38^ and in semantic dementia as discussed above.

As per left TLE, impoverished future thinking in right TLE is linked to poor performances on measures of lexico-semantic access and episodic autobiographic memory. Perhaps surprisingly, however, the future thinking deficit in left TLE is not greater than that seen in right TLE, despite the additional scene construction deficit in left TLE group. One possibility is that poor lexico-semantic access and episodic autobiographic memory dysfunction are the principal drivers of the future thinking impairment observed in both TLE groups, such that the scene construction deficit observed in left TLE does not additionally compound an already critically compromised future thinking system. Another possibility is that the right temporal lobe also makes a unique contribution to future thinking that is vulnerable to epilepsy-related disease, a contribution that was not otherwise assayed by the cognitive measures employed in the current study. The notion of a specific role for the right temporal lobe in future thinking is in keeping with the neuroanatomical model of prospection derived from an fMRI study of future thinking in healthy controls,^12^ and with the results of a case study of semantic dementia reporting that heightened activation of the right anterior hippocampus can seemingly compensate for atrophy in the left hippocampus during episodic future thinking.^39^ What is specific, however, about the known function of the right temporal lobe to future-orientated mental time travel?

A compelling hypothesis is that right TLE disrupts right-biased networks that subserve self-related processing, the retrieval of episodic autobiographical information, and creativity, and that a creative recasting of existing self-focused episodic information enables us to flexibly recombine our past experiences into novel imagined futures (see Fig. 2).^12,40–42^ These processes are thought to rely in part on a circuit local to the right temporal lobe comprising middle temporal gyrus, mesial temporal structure, fusiform gyrus and superior temporal gyrus.^42^ One interpretation of our data is that these creative processes needed to create a novel future scenario in the mind’s eye are disrupted by the right TLE. There is some precedence from case studies that neurological disease can alter creative episodic processes.^42,43^ While right TLE can certainly lead to reorganization of neurocognitive networks in the region,^44,45^ there is no compelling empirical evidence to date that TLE can alter creative capability specifically.^46,47^ This account, therefore, remains speculative, warranting further investigation.

### Role of semantic processing in future thinking

An interesting side finding of the current work was that intact future thinking in the healthy control group was associated with the integrity of their semantic autobiographic memory system; that is, their recall of personally relevant knowledge from across their lives such as an object or person’s name as well as their physical and sensory features. Given that the controls did not significantly differ from the people with epilepsy in terms of the *adequacy* of their semantic autobiographic memory, this indicates that neurologically normal individuals are able to rely on the efficient recollection of semantic memories to shape and flesh out future-oriented simulations in a way that people with epilepsy do not. This expands a finding of Lechowicz *et al.*^20^ whereby poor future thinking in the left TLE group specifically was linked to a dissolution in semantic fluency,^20^ replicated across both TLE groups in the current study. The contribution of autobiographic semantic memory and lexical access to future thinking observed in the current study is in keeping with a growing body of evidence that lexico-semantic networks crystallized around the anterior temporal lobes make a substantial contribution to future thinking.^8,40,48^

### Limitations and future directions

While our cohort is larger and more homogenous than any used previously in epilepsy,^20^ it nonetheless lacks the power to investigate any potential effects that might depend upon sub-lobar localization, which could provide important information regarding the neurocognitive architecture of future thinking. A longitudinal study of a large series of surgical cases could also be informative in this regard. Relatedly, the widely distributed nature of the neurocognitive system supporting future thinking involves areas beyond just the temporal lobe, and challenges us to examine whether extra-temporal or generalized epilepsies are also vulnerable to future thinking deficits.^5^ Future research would benefit from the inclusion of a richer assessment of executive abilities, given the importance of these processes to future thinking [refs]. A broadened set of measures would also be well suited to a factor analytic approach examining the degree to which future thinking reflects a unique cognitive operation.

### Clinical considerations for counselling TLE patients with impoverished future thinking

During medical consultations, most patients are assumed able to weigh up clinical advice by imagining and carefully considering a divergent array of possible lifestyle and health consequences of each treatment pathway presented to them. The results of the current study indicate that medical professionals ought to be mindful of how they deliver information to people with TLE in order to facilitate valid consent,^49^ given their apparent vulnerability to future thinking deficits.

A current impediment to the clinical translation of this study is that the measure of future thinking used is an experimental tool that is prohibitively time consuming to administer and score in clinical practice. An intriguing sub-finding of the present study is that the clinicians’ subjective judgement of future thinking integrity on a scale of 0–10 is highly correlated with the full-scale objective score. This raises the possibility that, for clinical purposes, future thinking can be evaluated validly and more easily than with the current system devised by Hassabis *et al*.^4^ Subsequent research could interrogate whether a greatly simplified administration and scoring system akin to the *Q*-score can be devised to improve the efficiency and clinical utility of the measure. A systematic revision could also consider measuring autobiographical recollection of past events and future simulation in a common manner, standardizing the assessment across temporal directions. As part of this psychometric process, future related scenarios could be redesigned to be more directly relevant to medical treatment decision-making, for instance, by explicitly asking patients to imagine and describe what their life might look like at a day-to-day level if they are seizure-free/able to drive/dysnomic/etc. This would form an adjunct to the clinical interview, where the patient is more informally questioned as to their future thinking capability and its day-to-day implications for them. The patient can then be scaffolded to more richly furnish these simulations in spite of their deficit in future thinking highlighted by the current study, so that they feel more confident in their long-term decisions.^50,51^

Such techniques would extend supportive counselling procedures already employed in many surgical programmes including our own^52^ and could help clinicians to efficiently identify and address overly vague, unrealistic or misguided expectations for surgery that are known to give rise to behavioural adjustment problems after the procedure.^53^

## Supplementary Material

fcac062_Supplementary_Data

## Abbreviations

AED =: antiepileptic drug
AMI =: autobiographical memory interview
BNT =: Boston Naming Test
COWAT =: Controlled Oral Word Association Test
IQ =: intelligence quotient
RAVLT =: Rey Auditory-Verbal Learning Test
TLE =: temporal lobe epilepsy
