# Supplementary material for: Compromised future thinking: another cognitive cost of temporal lobe epilepsy
Source: Brain Commun. 2022 Mar 19;4(2):fcac062. doi: 10.1093/braincomms/fcac062 (PMC8963290; doi:10.1093/braincomms/fcac062)
Supplement: fcac062_Supplementary_Data [file fcac062_supplementary_data.docx]

**Compromised future thinking: another cognitive cost of temporal lobe epilepsy (Rayner et al)**

***Supplementary Material***

**Subscale score analyses for the Scene Construction Task**

Secondary ANOVA showed that both Left and Right TLE are worse than Controls on the Content (T) scale (P < .01 in each case) and the Quality (Q) scale (P < .001 in each case); and that Left TLE is worse than both Right TLE and Controls on the Spatial Coherence (C) scale (P < .03 in each case) with Right TLE and Controls comparable to one another. There were no group differences on metrics of subjective Salience (S) or Presence (P; i.e., sense of reexperiencing). This pattern of results is shown in the boxplots below:


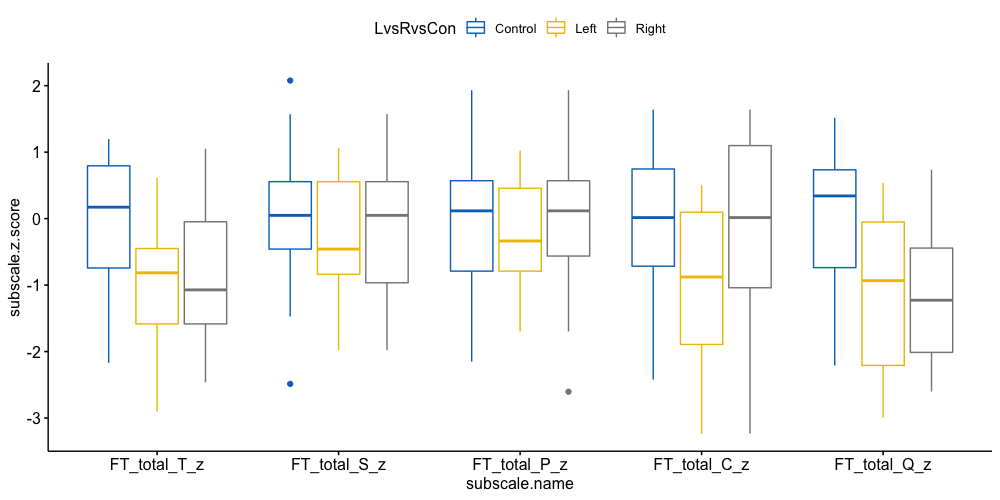


We also include an interaction plot below.

*(Note that for ease of comparison, scores in each group on each scale are expressed in z units relative to the control group scores on that scale):*


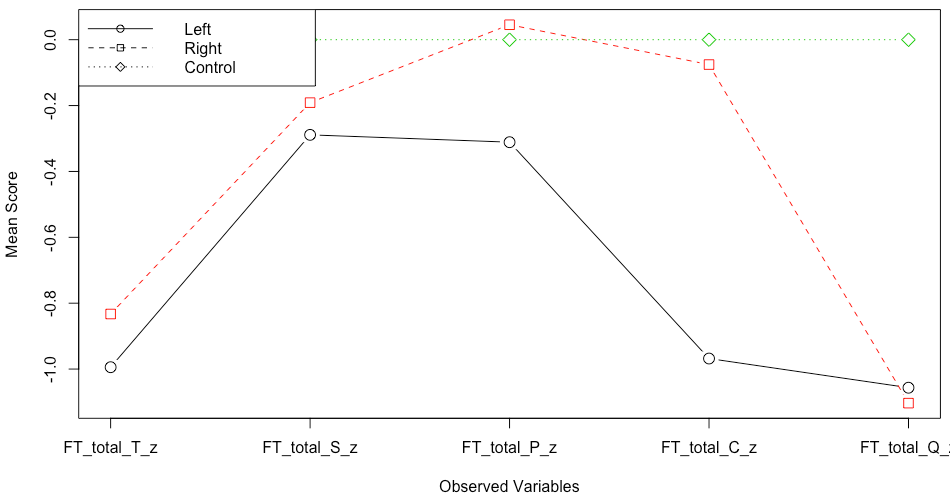


Together, these analyses indicate that people with TLE do worse on the future thinking task because of poor scores on the Content and Quality scales, with Left TLE also producing poor Spatial coherence scale scores.
